# Data on High Resolution Melting (HRM) and phylogenetic analysis of *P. ovale wallikeri* and *P. ovale curtisi*

**DOI:** 10.1016/j.dib.2019.103937

**Published:** 2019-04-23

**Authors:** Aline Lamien-Meda, Hans-Peter Fuehrer, Harald Noedl

**Affiliations:** a Institute of Specific Prophylaxis and Tropical Medicine, Medical University of Vienna, Austria; b Institute of Parasitology, University of Veterinary Medicine, Vienna, Austria

## Abstract

High Resolution Melting (HRM) analysis is a post-PCR analysis method used for identifying genetic variation in nucleic acid sequences. These data are presenting the identity of the 33 samples used for a qPCR-HRM and a nested snapback methods validation. In addition we are presenting the high resolution melting profiles of *P. ovale curtisi* (Poc) and *P. ovale wallikeri* (Pow) in the following conditions: after a direct qPCR run and after a nested snapback run. The qPCR-HRM of artificial mixture of Poc and Pow plasmids (200 copies/μl, each) at different proportions are showing the melting pattern of co-infections with both species. The sequencing methodology of the c*lpc* gene fragment of 12 randomly selected samples is described and their likeness to published sequences is shown in a maximum likelihood tree. “Novel high resolution melting and snapback assays for simultaneous detection and differentiation of Plamodium ovale spp.” [1].

**Table:** Specifications Table

Subject area	*Biology*
More specific subject area	*High resolution melting Assay, Phylogenetic Analysis*
Type of data	*Table, text file, figure*
How data was acquired	*LightCycler 480 qPCR system HRM assays (Roche Diagnostics GmbH)*
Data format	*Analyzed*
Experimental factors	*Melting program: Denaturation of the PCR product at 95°C for* 1°min *followed by a cooling to 50°C for* 1°min *and a continuous heating at 0.*02°C/s*; Fluorescence acquisition was done from 50°C to 80°C.* *The clpc fragments and their phylogenetic analysis processes are described in text file.* *Artificial mix melting profiles of P. ovale curtisi (200 copies/μl) and P.ovale wallikeri (200 copies/μl) at the following proportion: 8/2; 7/3; 5/5; 3/7; 2/8*
Experimental features	*The separated melting curves of both P. ovale species are showing the method accuracy and the difference between the melting temperatures. The Tms of artificial mixture of both species plasmids at different proportion are presented.* *The sequencing is providing additional clpc fragments for the gene analysis, and the phylogenetic analysis is comparing our sequences to published sequences*
Data source location	*Vienna, Austria*
Data accessibility	*All data are presented in this article*
Related research article	*Lamien-Meda, A., Fuehrer, H.P. and Noedl, H., 2019. Novel High Resolution Melting (HRM) and Snapback Assays for Simultaneous Detection and Differentiation of Plasmodium ovale spp. Acta Tropica, 192, 75–81*[1].

**Table:** 

**Value of the data** • The data present the complete list of samples used to validate the qPCR- HRM and snapback assays with their microscopy, nested PCR and PrimerDesign qPCR genotyping data. • A phylogenetic tree presents the classification of the *Clpc* gene sequences of the selected *P ovale* samples, comparatively to those of other studies. • The data show the separated melting curves of the qPCR-HRM assay of the two *P. ovale* species. Differences between the melting temperatures (Tm) of the qPCR-HRM and the snapback assays are presented in boxplots. • The Tm profiles of Poc and Pow using artificial mixture of Poc/Pow at the proportions of 8/2; 7/3; 5/5; 3/7; 2/8, are presented.

## 1 Data

**Fig. 1:**
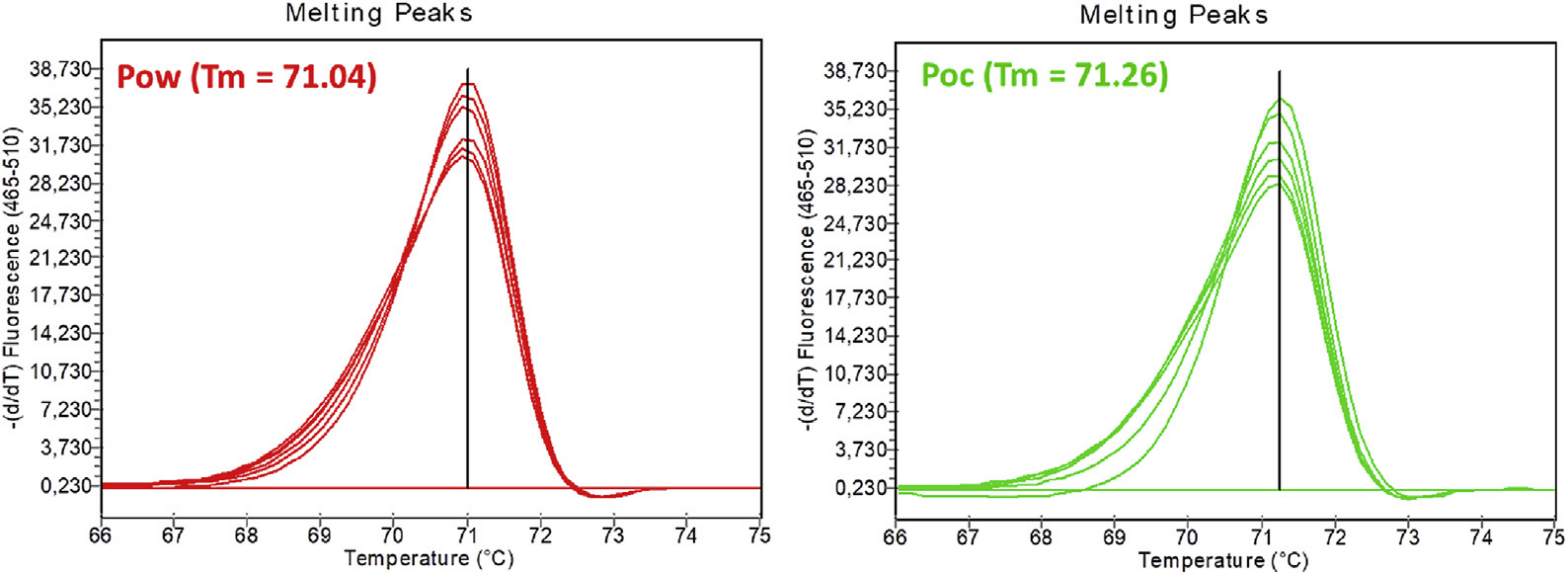
Separated melting curves of Pow (red) and Poc (green). The lines at the middle of the curves are presenting the accuracy of the Tm with equal Tm value of the sample.

**Fig. 2:**
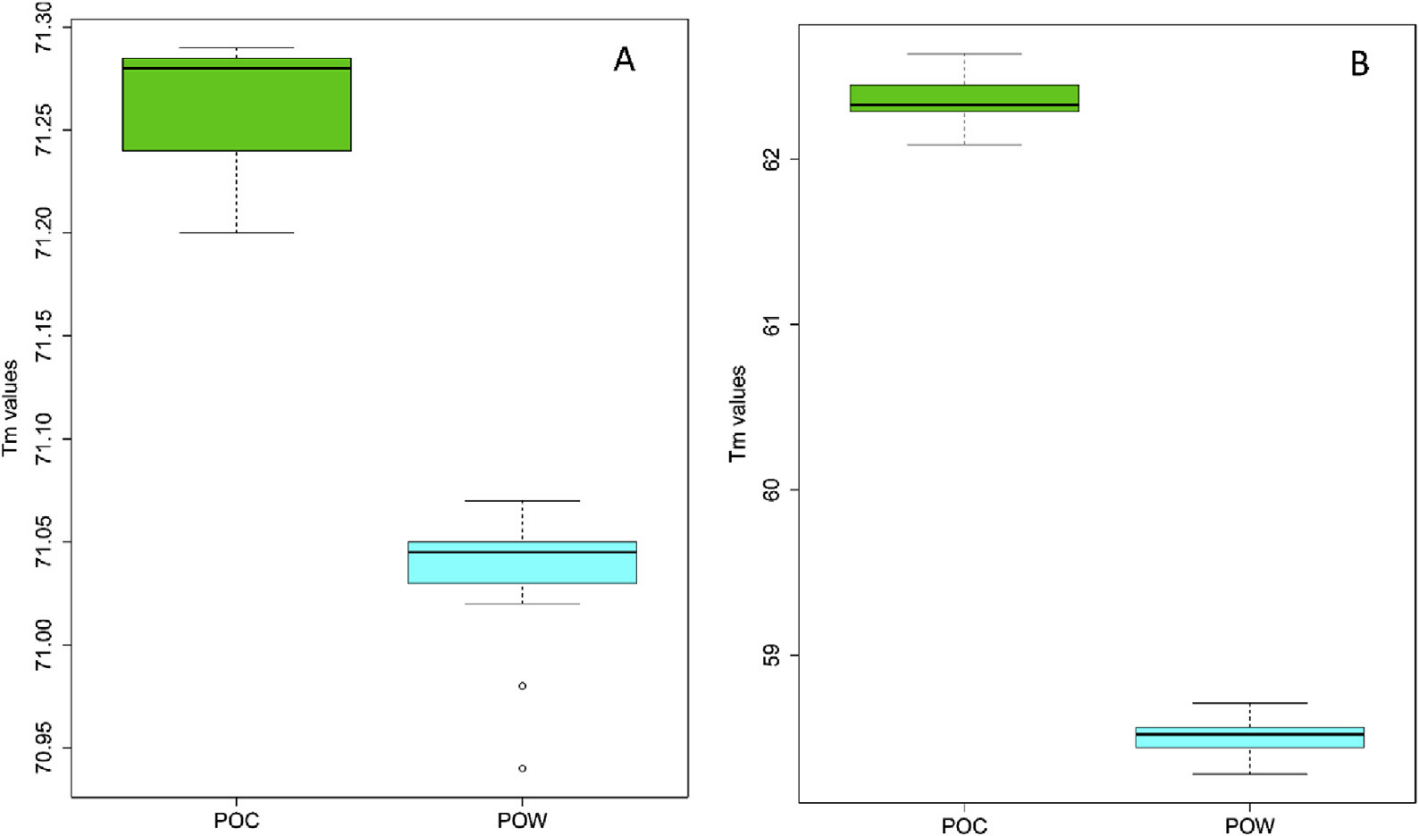
Boxplots of the melting temperatures (Tm) of *P. ovale curtisi* (green) and *P. ovale wallikeri* (blue) genotypes (A), and of the snapback stem genotypes (B). The box indicates the likely range of melting temperature variation with the median as a segment inside the box. Whiskers above and below show the Tm range and outliers are represented in circle. The Tm difference between Poc and Pow were ΔTm = 0.2°C (A) and ΔTm = 3.74°C (B). The Welch's unequal variances *t*-test showed that Tm of the amplicon Poc and Pow were significantly different for both HRM and snapback genotyping (p-value < 2.2e-16).

**Fig. 3:**
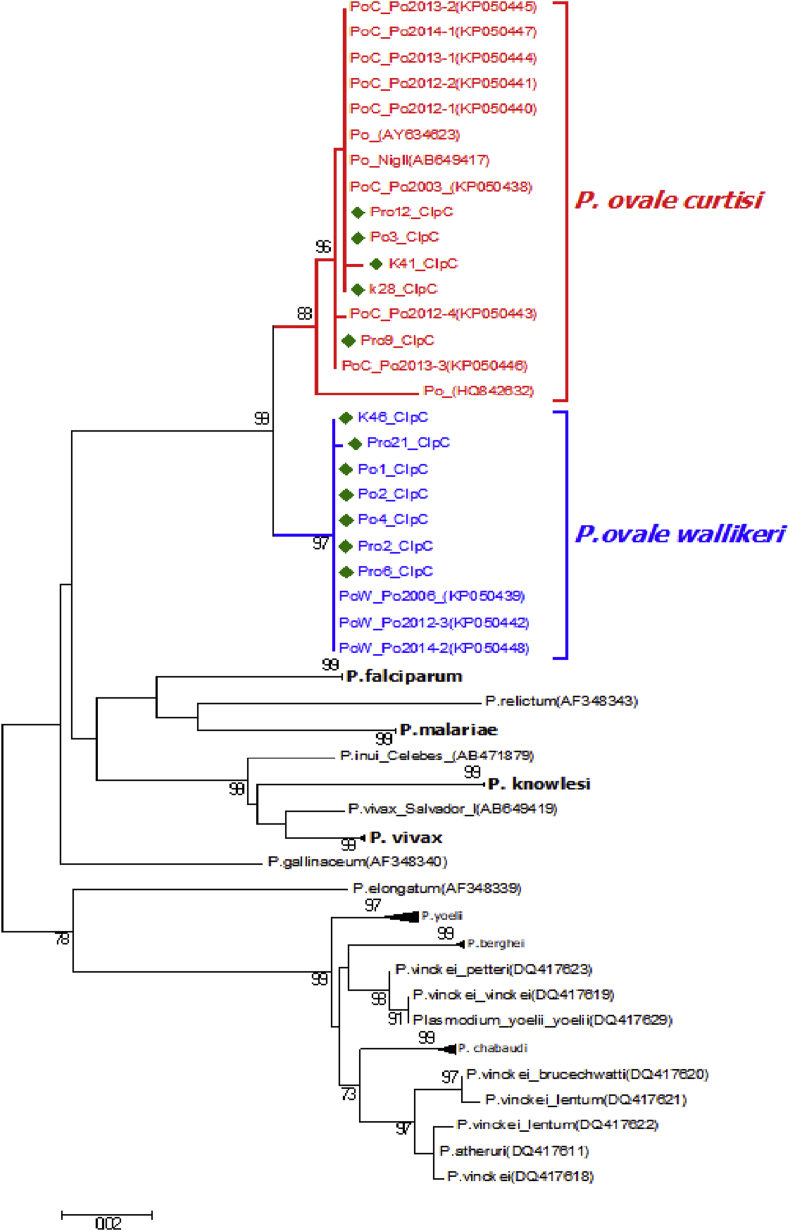
Maximum likelihood tree of 61 *Plasmodium sp. clpc* gene. The *clpc* gene fragment of 12 samples was amplified, sequenced, aligned and compared to those from other studies [2], [3], [4] in order to confirm their identity. The green diamonds are indicating the selected 12 samples. The scale bar shows the number of nucleotide substitutions per site.

**Fig. 4:**
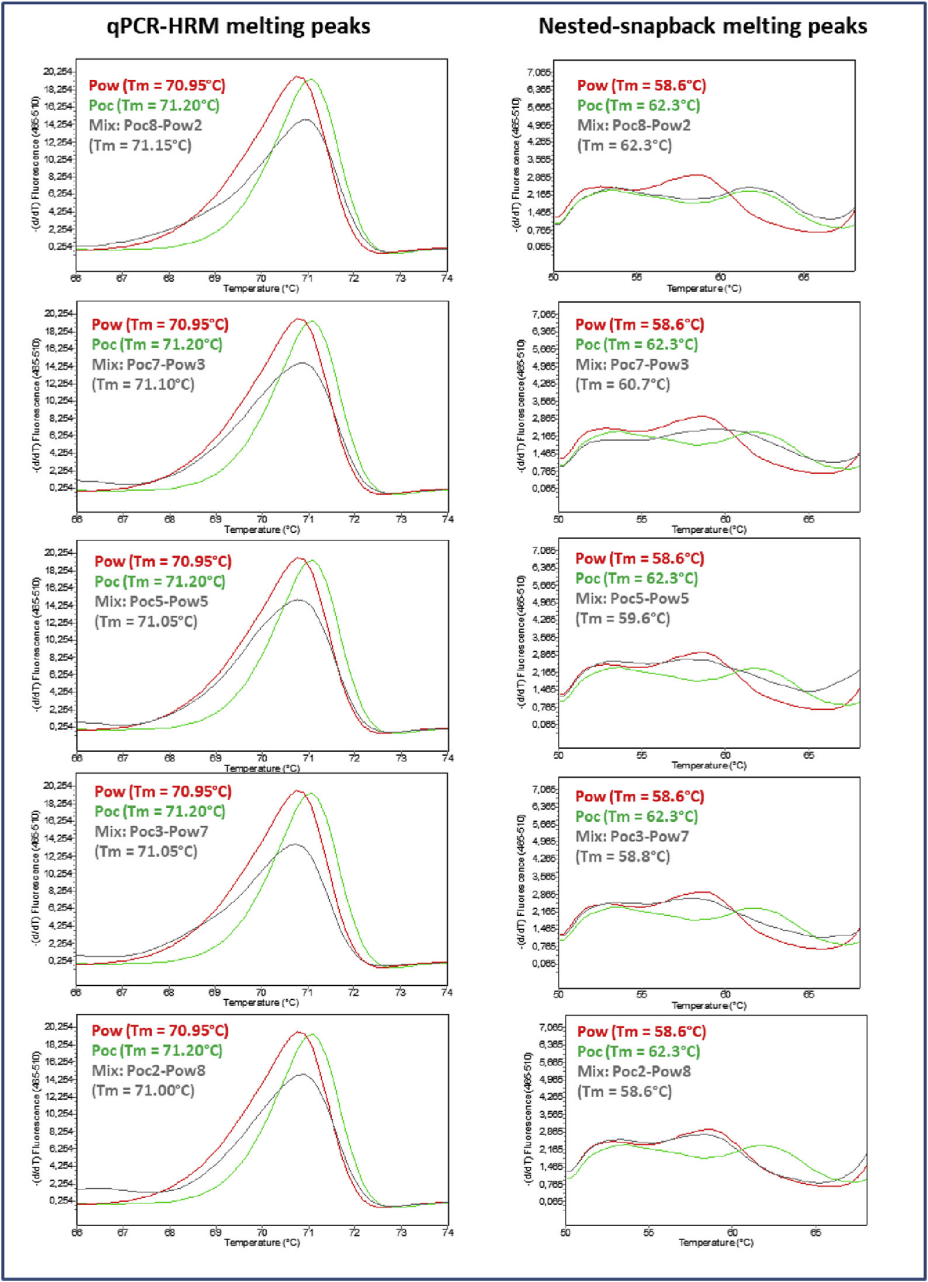
qPCR-HRM and Nested snapback melting curves obtained from artificial mixes from Poc and Pow. The following five (5) artificial mixes with various ratios were done: 8/2, 7/3, 5/5, 3/7, 2/8, for respectively Poc/Pow. All mix samples produced an intermediate Tm (71.07 ± 0.05°C) with the qPCR-HRM assay. For the snapback reaction, the intermediate Tm values were 60.0 ± 1.5°C.

This report is presenting in Table 1 the detailed list of all thirty three (33) samples used to validate a qPCR-HRM and a nested snapback methods developed for *P. ovale* species differentiation [1]. Table 1 is presenting the origin of samples and the results of different genotyping methods (microscopy, nested-PCR,qPCR-HRM and PrimerDesign kit). The *P. ovale clpc* gene fragments of twelve (12) samples selected randomly were sequenced. The obtained sequences were used for a phylogenetic reconstruction together with previously published *P. ovale clpc* sequences (Fig. 3). The PCR and HRM melting curves of *P. ovale curtisi* (Poc) and *P. ovale wallikeri* (Pow) are shown in Fig. 1 and the differences between the melting temperatures (Tm) are presented in Fig. 2. The nested snapback ΔTm was 3.74°C and that of the direct qPCR-HRM was 0.2°C. Intermediate Tm values of 71.07 ± 0.05°C (for qPCR-HRM reaction) and 60.0 ± 1.5°C (for nested snapback reaction) were observed with artificial mix of Poc/Pow at the proportions of 8/2, 7/3, 5/5, 3/7 and 2/8 (Fig. 4).

**Table 1:** *P. ovale curtisi* (Poc) and *P. ovale wallikeri* (Pow) samples tested with the qPCR-HRM and snapback assays with their microscopy, nested PCR and PrimerDesign qPCR genotyping.

No.	Sample ID	Origin	Microscopy^a^	Parasite density/μl^c^	Nested PCR	qPCR-HRM genotyping	Snapback genotyping	PrimerDesign Kit^b^
1	F3	Ethiopia	Pv	–	Pow	Pow	Pow	Po
2	T52	Ethiopia	n.d.	–	Pow	Pow	Pow	Po
3	K21	Ethiopia	Pv	–	Pow	Pow	Pow	Po
4	K28	Ethiopia	Pf	–	Poc	Poc	Poc	Po
5	K41	Ethiopia	Pv	–	Poc/Pf	Poc	Poc	Po
6	K46	Ethiopia	Pv	–	Pow	Pow	Pow	Po
7	Pro2	Ethiopia	Pv	4920	Po^b^	n.d.	Pow	n.d.
8	Pro4	Ethiopia	Pv	5600	Po^b^	n.d.	Pow	n.d.
9	Pro6	Ethiopia	Pv	15200	Po^b^	Pow	Pow	Po
10	Pro9	Ethiopia	Pv	4920	Pf/Po	Pow	Pow	Po
11	Pro12	Ethiopia	Pv	6000	Po^b^	Poc	Poc	Po
12	Pro21	Ethiopia	Pv	7200	Pf/ova	Pow	Pow	Po
13	5	Ethiopia	n.d.	–	Poc	Poc	Poc	n.d.
14	SG9255	Ethiopia	n.d.	–	Pow	Pow	Pow	Po
15	Po1	Bangladesh	Pv	2240	Pow	Pow	Pow	Po
16	Po2	Bangladesh	Pm	6680	Pow	Pow	Pow	Po
17	Po3	Bangladesh	Pm	2600	Pow	Pow	Pow	Po
18	Po4	Bangladesh	Pv	280	Poc	Poc	Poc	Po
19	Po5	Bangladesh	Pv	120	Pow	Pow	Pow	Po
20	Po7	Bangladesh	Pv	440	Pow/pf	n.d.	Pow	n.d.
21	Po8	Bangladesh	Pv	320	Pow/pm/pf	Pow	Pow	Po
22	Po9	Bangladesh	Pf	14520	Pow/Pf	n.d.	Pow	n.d.
23	Po10	Bangladesh	Pf+Pv	480	Poc/Pf/Pv	Poc	Poc	Po
24	Po11	Bangladesh	neg	–	Pow	Pow	Pow	Po
25	Po12	Bangladesh	neg	–	Poc	Poc	Poc	Po
26	Po14	Bangladesh	neg	–	Poc	Poc	Poc	Po
27	Po15	Bangladesh	neg	–	Poc	Poc	Poc	Po
28	Po16	Bangladesh	neg	–	Poc/Pf	Poc	Poc	Po
29	Po17	Bangladesh	Pf	3080	Poc/Pf/Pm	Poc	Poc	Po
30	Po18	Bangladesh	neg	–	Poc/Pf	Poc	Poc	Po
31	Po20	Bangladesh	neg	–	Poc/Pf/Pv/Pm	Poc	Poc	Po
32	Po21	Bangladesh	neg	–	Pow/pf/pv/pm	Pow	Pow	Po
33	Po22	Bangladesh	neg	–	Pow/Pf/Pv/Pm	Pow	Pow	Po

n.d. (not detected); Po (*Plasmodium ovale*); Pf (*Plasmodium falciparum*); Pv (*Plasmodium vivax*); Pm (*Plasmodium malariae*). Further information of Bangladeshi ovale samples can be found in Ref. [5].

a *P. ovale* spp. was not known to be endemic in the sampling areas.

b *P. ovale* species was not characterized.

c Parasite density/μl was not evaluated for all samples.

## 2 Experimental design, materials and methods

### 2.1 qPCR-HRM and nested snapback assays

The melting curves of Poc and Pow in Fig. 1 and the melting temperatures (Tm) shown in Fig. 2 were obtained by PCR and high-resolution melting reactions using a Roche LightCycler 480 qPCR system (Roche Diagnostics GmbH, Mannheim, Germany). Details of the qPCR-HRM and the snapback assays are given in Ref. [1].

### 2.2 Clpc gene fragments amplification and sequencing

A fragment of the *clpc* gene (640 bp) of 12 randomly selected samples was amplified by PCR from total genomic DNA using primers previously designed by Perkins et al. [2]: Perkins_*clpc*F (5′-GGTAAAACTGAATTAGCAAAAATATTA-3′) and Perkins_*clpc*R (5′-GGACGAGCTCCATATAAAGGATT-3′). The PCR reaction was performed with initial denaturation at 95°C (4 min) and 40-cycles of denaturation (95°C, 20 sec), annealing (50°C, 30 sec) and extension (72°C, 50 sec). The PCR products were separated by electrophoresis in a 2% agarose gel. The PCR positive products were sequenced commercially by LGC Genomics.

The sequences were edited using Vector NTI version 11.5 and BioEdit software package version 7.2.6. Multiple sequence alignments were performed using the clustal W algorithm, as implemented in MEGA 7, to compare the obtained sequences to a set of published *P. ovale clpc* sequences from other studies [2], [3], [4] retrieved from GenBank (Accession numbers KP050438 – KP050448, AB649417, AY634623, HQ842632, KX611805, LT594596, LT5994519).

### 2.3 Phylogenetic reconstructions

For phylogenetic reconstructions, the most appropriate model of molecular evolution was determined by the Akaike Information Criterion (AIC) using MEGA7. Maximum likelihood (ML) analyses with 1000 bootstrap replicates were performed using the program MEGA7 with the predetermined model of molecular evolution (GTR+I+G for both datasets) using all sites. All the *Plasmodium* species clustered separately with strong bootstrap support. Additionally, the *P. ovale wallikeri* and *P. ovale curtisi* formed two distinct sub-clusters with strong bootstrap support. Among the 12 samples that were sequenced, 7 (Po1, Po2, Po4; K46, Pro2, Pro6, Pro21) were clustered with *P. ovale wallikeri* and 5 (Po3, Pro9, Pro12, K28, K41) were clustered with *P. ovale curtisi*.
